# Automated Early Detection of Skin Cancer Using a CNN-ViT-Attention-Based Hybrid Model

**DOI:** 10.3390/biomedicines14030583

**Published:** 2026-03-05

**Authors:** Zekiye Kanat, Merve Kesim Onal, Harun Bingol, Serpil Sener, Engin Avci, Muhammed Yildirim

**Affiliations:** 1 Department of Dermatology, Inonu University, Malatya 44280, Türkiye; zekiye.kanat@inonu.edu.tr (Z.K.); serpil.sener@inonu.edu.tr (S.S.); 2 Department of Computer Engineering, Malatya Turgut Ozal University, Malatya 44210, Türkiye; merve.kesim@ozal.edu.tr; 3 Department of Software Engineering, Malatya Turgut Ozal University, Malatya 44210, Türkiye; harun.bingol@ozal.edu.tr; 4 Department of Software Engineering, Firat University, Elazig 23119, Türkiye; enginavci@firat.edu.tr; 5 Department of Artificial Intelligence and Data Engineering, Firat University, Elazig 23119, Türkiye

**Keywords:** attention, classifiers, CNN, skin cancer, ViT

## Abstract

**Background/Objectives**: Skin cancer is a very serious disease. There is a risk that the cancer will spread to other parts of the body as the cancerous tissue deepens. For this reason, early diagnosis is important because it allows for early initiation of treatment. This study proposes a hybrid model for the early diagnosis of skin cancer. **Methods**: The proposed model was developed using Convolutional Neural Networks (CNNs), Vision Transformer (ViT) architectures, and the k-Nearest Neighbors (KNN), Support Vector Machine (SVM), Naive Bayes (NB), Neural Network Classifiers, Decision Tree (DT), and Logistic Regression (LR) classifiers. Furthermore, the proposed model was fine-tuned to improve its disease diagnosis. Two attention mechanisms, channel and spatial, were used together in the proposed model. The HAM10000 dataset was used during the experiments. Class weighting was performed to ensure class-based balance in the dataset. **Results**: The proposed model was also compared with the CNN and ViT architectures frequently used in the literature. Among these models, the highest accuracy value of 95.1% was obtained with the proposed model. **Conclusions**: It is considered that the proposed model can be used as a decision support system for dermatologists in the diagnosis of skin cancer.

## 1. Introduction

Skin cancer is a very common type of cancer worldwide [1]. Skin cancer is less common in people with darker skin compared to those with lighter skin [2]. However, it is known that mortality rates due to the disease are higher in people with darker skin. Individuals who are directly exposed to sunlight have a higher probability of developing skin cancer. However, sunlight is not the only cause of skin cancer. The patient’s genetic predisposition and other environmental factors are also very effective in the onset and development of this disease [3,4]. If the disease is not diagnosed early and treatment is not started, the excessive proliferation of cancerous cells and their spread to other parts of the body can lead to death [5].

Skin cancer is usually diagnosed by examining a biopsy taken from the suspicious tissue. This method is a lengthy and costly process [6]. In recent years, with the advancement of technology, computer-aided systems have begun to be used effectively in many fields such as transportation [7], health [8], the pharmaceutical industry [9], aviation [10], and agriculture [11]. They are also used effectively in the diagnosis and detection of diseases. In addition to costly procedures like biopsies, computer-assisted imaging systems can help specialists diagnose skin cancer. Differentiating between subtypes of skin cancer is a highly specialized task. Accurate classification of lesions is of paramount importance in diagnosing the disease [12]. Manual systems are generally time-consuming, expensive, and prone to error because they are performed by humans. Furthermore, there is a shortage of specialists in rural areas. For these reasons, computer-assisted systems are crucial, both to lighten the workload of specialists and allow them to treat more patients, and to enable early detection of the disease and the initiation of necessary treatment [13].

Deep learning methods are among the most widely used computer-aided systems. Deep learning techniques, a subfield of artificial intelligence, are known to achieve highly successful results in image classification problems. This study utilizes the publicly available HAM10000 dataset. A transformer-based hybrid model is proposed as a decision support system for the early diagnosis of skin cancer. In the proposed model, the most important features of disease images are extracted using both CNN-based and transformer-based methods. Fine-tuning was performed on the proposed model to improve its performance and enable better training. Two different attention mechanisms were applied to the feature map obtained by concatenating the features obtained from CNN and ViT architectures. This allowed the model to focus on diseased areas in the image. The number of images in the classes in the HAM10000 dataset shows an unbalanced distribution. To correct this imbalance, a class weighting method was applied. As a result of the experiment, it is evaluated that the hybrid model proposed in this study can be used by experts as a decision support system in the diagnosis of skin cancer.

### 1.1. Contribution and Novelty

This study involved a detailed literature review. Many studies were observed to focused on the diagnosis and classification of skin cancer. The contributions of this study to the wider literature are as follows:

A hybrid CNN-ViT and attention-based model is proposed for use in the early diagnosis of skin cancer. The proposed model was tested on the KNN, SVM, NB, NN, DT, and LR classifiers. 

In the first stage of the proposed model, feature extraction was performed using ConvNeXt-Base and Swin-Base transformer architectures. These features were passed through two different attention modules, then classified in classifiers after class weighting.

Fine-tuning was applied to the proposed model to improve skin cancer diagnosis accuracy.

The proposed model achieved a highly competitive accuracy value (Test accuracy: 95.1%) on the HAM10000 dataset.

### 1.2. Releated Works

There are studies in the literature regarding skin cancer. Dildar et al. stated that skin cancer causes irreparable damage to the skin surface. They noted that skin cancer spreads to other organs of the body more slowly compared to other types of cancer. They emphasized that early diagnosis is extremely vital at this point [14].

Hosny et al. used CNN architectures in the diagnosis of skin cancer. They conducted experiments on the Ph2 dataset. Since this dataset contains a total of 200 color images, they amplified the dataset 55 times to increase the number of images. They stated that they obtained 55 new images of each image. While the accuracy rate obtained in experiments with the original dataset was 80%, the accuracy rate obtained after the augmentation process was stated to be 98.61% [15]. Although data augmentation techniques are known to have a positive effect on model learning, increasing the dataset by 55 times can lead to overlearning; therefore, these techniques should be used within certain limits. Furthermore, the augmented data should only be used in the training set and not in the test set.

Gouda et al. stated that they used the two-class ISIC2018 dataset during experiments to classify skin cancer. They stated that they increased the number of images in the dataset to 3533 using augmentation techniques. Furthermore, the researchers stated that they improved the skin cancer lesion images in the dataset using the ESRGAN method. This method aimed to remove rough edges. They stated that they achieved an accuracy rate of 85.8% using the InceptionV3 architecture [16]. The ISIC2018 dataset contains information about the presence or absence of diseases because it is divided into two classes. However, for artificial intelligence models to effectively assist physicians, it is important to diversify the diseases and provide information about the specific type of disease. From this perspective, it is believed that the dataset needs to include more classes.

Gururaj et al. used the seven-class HAM10000 dataset for skin cancer diagnosis during their experiments. The researchers stated that they achieved an accuracy of 91.2% with DenseNet169 using a sample reduction technique. The imbalance in the number of images across classes in the dataset and the use of data reduction techniques to address this imbalance significantly impacted the model’s performance [17].

Jinnai et al. reported that in their study, they extracted a total of 5846 images of pigmented skin lesions from 3551 patients. In their research on skin cancer, they noted that CNN-based models, especially in recent years, can classify skin cancer with an accuracy close to that of dermatologists. In their study, they classified skin lesions with 86.2% accuracy [18].

Kousis et al. stated that they used the HAM10000 dataset in their experiments to classify skin cancer. They stated that they applied data enhancement and fine-tuning operations to eliminate the high similarities of skin lesions in this well-known dataset. In the dataset they tested with eleven different CNN architectures, the highest performance was achieved by the DenseNet169 architecture with an accuracy of 92.25% [19].

Tembhurne et al. stated in their study that skin cancer is a very common type of cancer and that 6 out of every 7 cases, especially melanoma, result in death. The researchers stated that they used manual and automatic feature extraction methods together in their study and thus achieved a classification with an accuracy rate of 93%. They stated that they used Logistic Regression (LR) and Linear Support Vector Machine (LSVM) techniques as machine learning classifiers and the VGG19 technique as a deep learning method [20].

Wang et al. used the publicly available ISIC2019 dataset during experiments to classify skin cancer. They stated that they achieved an accuracy rate of 90.67% with the VGG deep model. Researchers highlighted the importance of frozen layers and data enhancement among the factors affecting the model’s performance. Researchers have also stated that the detection of this highly lethal type of melanoma using automated systems is important [21]

Balambigai et al. stated that they used CNN architectures to identify skin cancer early. They used the HAM10000 dataset during the experiments. The researchers emphasized that hyperparameter selection is quite important in the performance of CNN architectures. For this, they stated that they used the random search optimization technique during the experiments. As a result of the experiments, they stated that they achieved an accuracy rate of 77.17% [22].

### 1.3. Organization of Paper

The article is organized as follows: Section 2 describes the dataset to be used in the early diagnosis of skin cancer, the CNN and ViT architectures, attention structures, class weighting operations to prevent unbalanced class distributions, fine-tuning operations applied to improve the learning of the proposed model, and the proposed model itself. Section 3 presents the results obtained from pre-trained models and the proposed model. Section 4 examines the performance of the proposed model and addresses the limitations of the study. The final section contains the conclusion of the article.

## 2. Materials and Methods

The main tools and methods used in the development of the proposed model are summarized in this section.

### 2.1. Dataset

The dataset used in the study is the HAM10000 dataset, which is the most well-known dataset used in many studies for the diagnosis and detection of skin cancer. This dataset contains a total of 10,015 images consisting of 7 classes. These classes are vascular lesions (VASC), dermatofibroma (DF), basal cell carcinoma (BCC), melanoma (MEL), melanocytic nevi (NV), benign keratosis-like (BKL) lesions, and actinic keratoses (AKIEC) [23]. Class-wise distribution of the dataset is shown in Table 1.

**Figure 1:**
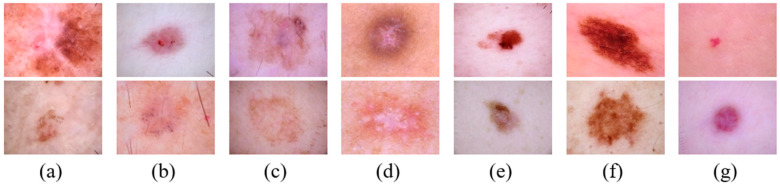
Sample images from the HAM10000 dataset (**a**) AKIEC (**b**) BCC (**c**) BKL (**d**) DF (**e**) MEL (**f**) NV (**g**) VASC.

HAM10000 was created by two different sources: the ViDIR Group of the Dermatology School at the University of Vienna in Austria, and Cliff Rosendahl and the Queensland University Medical School of Australia [23]. Sample images from the dataset are presented in Figure 1.

### 2.2. Class Weight

The class imbalance problem (imbalanced datasets), frequently encountered in medical image classification studies, causes models to overfit the majority class and ignore the critically important minority class. This situation increases the tendency of the model to misclassify the minority class while achieving high accuracy in the majority class [24].

As shown in Table 1, the HAM10000 dataset used in this study also has significant inter-class imbalances. There is a total of 6705 samples in the NV class, while there are only 115 samples in the DF class. While applying traditional data augmentation techniques to unbalanced datasets helps increase the diversity of minority classes, synthetic samples generated, particularly in medical image classification studies, often fail to adequately represent the complex and low-variance nature of minority classes [25]. Therefore, class-weighted loss functions are frequently preferred in the literature to mitigate the class imbalance problem [26,27,28,29]. Yin Cui et al. proposed a class-balanced loss function based on the effective number of samples to solve the class imbalance problem [29]. In this approach, the weight for each class $W_{i}$ is calculated as follows:

$$W_{i}=\frac{1-\beta }{1-\beta^{n_{i}}}$$

where $n_{i}$ denotes the number of samples in class i, and β is a hyperparameter $\left(\beta \in \left[0,\;1\right)\right)$ that controls the growth of the effective number of samples.

Similarly, in this study, a class weight was used in all training sessions where the class samples were weighted according to the effective number of samples. This increased the contribution of minority class samples and aimed to improve the model’s performance in distinguishing these classes.

### 2.3. Overall Proposed Model

In this paper, a hybrid model was proposed for the early detection of skin cancer. The first stage of the developed model is the feature extraction stage. For this purpose, feature extraction was first performed with the EfficientNet-b0 [30], Resnet50 [31], ConvNeXt_Base [32], Inception_Resnet_v2 [33], DenseNet 121 [34], Inception v3 [35], VGG19 [36], Xception [37], ViT B-16 [38], ViT B-32 [39], DeiT B-16 [40], Swin Base [41] architectures, which are accepted in the literature. Then, two models that gave the highest accuracy values on the HAM10000 dataset were determined from among the pre-trained ViT and CNN architectures and were selected to be used in the subsequent stages. Both architectures were subjected to fine-tuning to enable them to learn the specific features of the HAM10000 dataset, achieving more successful feature extraction than the transfer learning approach using pre-trained weights on ImageNet. Class weights were also applied during the fine-tuning phase to address the inter-class imbalance problem in the dataset. In the fine-tuning phase, the selected models were trained for five epochs, and the updated weights from the epoch where the best performance was obtained were saved for use in subsequent stages. The specific hyperparameters and implementation settings utilized during this fine-tuning phase are provided in Table 2. The choice of 5 epochs for the fine-tuning phase was determined based on the rapid convergence observed during the preliminary experiments. Given that the backbone architectures (ConvNeXt-Base and Swin-Base) were pre-trained on the large-scale ImageNet dataset, they already possessed robust feature extraction capabilities. By employing a relatively low learning rate of 1 × 10^−5^ and an automated scheduler, the models achieved a stable state quickly. This limited training duration also acted as a preventive measure against overfitting, particularly given the dataset size and class imbalance.

Then, the ConvNeXt and Swin-Base Transformer architectures were retrained with these fine-tuned updated weights. Feature vectors were extracted from the average pooling layer of both models. A 1024 feature vector was concatenated from each model to create a combined feature vector of 2048 dimensions.

The generated 2048-dimensional feature vector was fed into Transformer-based Spatial attention [42] and Squeeze-and-Excitation (SE)-based Channel attention [43] mechanisms, respectively, to ensure the model focuses on critical features. Spatial attention, used in the first stage, unlike classical convolutional approaches, focuses on spatial importance through a Transformer Encoder structure, modeling the global relationship between all locations. Channel attention, used in the second stage, learns the relationships between different channel information and determines which channel contributes more to the classification task, adjusting the weight for each channel.

**Figure 2:**
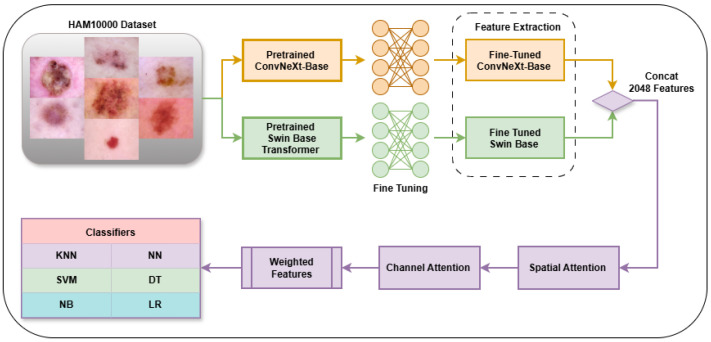
Flowchart of the proposed model.

Following these sequential attention mechanisms, the resulting Weighted Features vector model was fed as input to six different traditional classifiers for evaluation. In this context, k-nearest neighbors (KNN) [44], Support vector machine (SVM) [45], Naive Bayes (NB) [46], Neural Network (NN) [47], Decision Tree (DT) [48] and Logistic Regression (LR) [49] algorithms were applied, and the performance of each classifier was compared. To ensure the reproducibility of the study and maintain consistent results across different runs, all data partitioning and model initialization processes were conducted using a fixed random seed. For the classification stage, the machine learning models were configured with specific hyperparameters as follows: the KNN classifier was configured as Fine KNN (k = 1, Euclidean distance metric), the SVM classifier as Cubic SVM (polynomial kernel of degree 3), the Neural Network classifier as Medium Neural Network (single hidden layer with 25 neurons), the Naive Bayes classifier as Kernel Naive Bayes, the Logistic Regression classifier as Efficient Logistic Regression, and the Decision Tree classifier as Fine Tree. The proposed model is given in Figure 2.

The developed model was also compared with different CNN and ViT methods accepted in the literature.

## 3. Results

This section presents the results of experimental studies conducted to evaluate the proposed model. The model’s performance was measured using standard metrics such as accuracy, recall, precision, and F1-score [50]. In all architectures used, class samples were weighted according to the number of classes using class weight.

Experiments were conducted in an environment with a 12th Gen Intel^®^ Core™ i7-12700H (2.70 GHz) processor, 16 GB RAM, and an NVIDIA GeForce RTX 3070 graphics processing unit with 8 GB VRAM. On the software side, Python 3.12.3 and CUDA Toolkit 12.6 were preferred. In addition, 80% of the HAM10000 dataset was used for training and 20% for testing to evaluate the model.

### 3.1. Results of Pre-Trained Models

**Figure 3:**
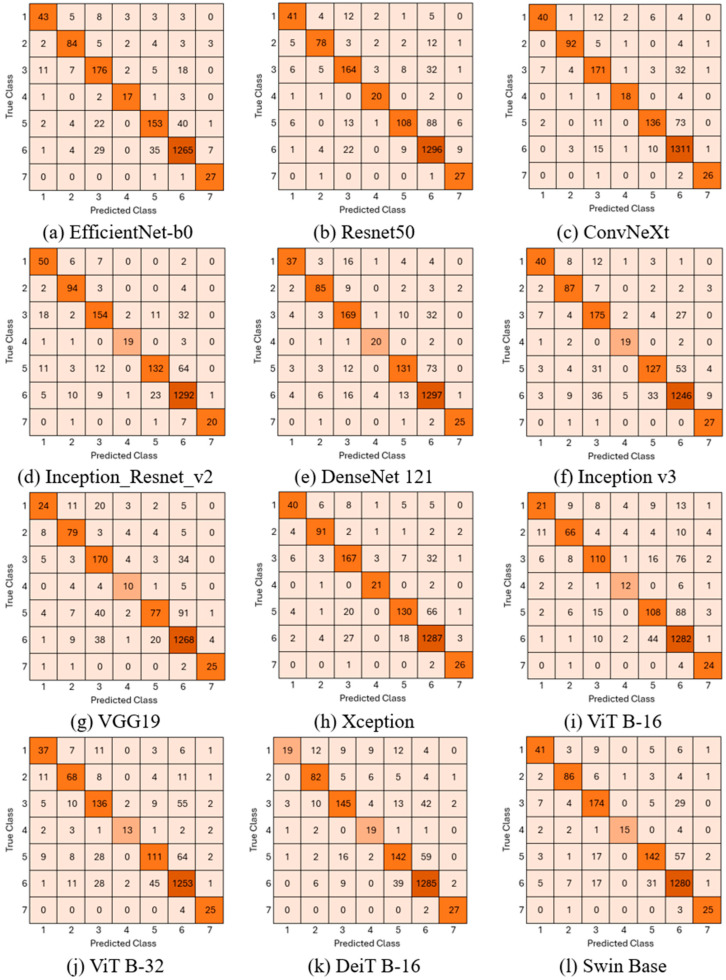
Confusion matrices of Pre-Trained models.

To determine the most suitable deep learning architecture for the proposed hybrid structure, various pre-trained CNN and Transformer models were evaluated on the HAM10000 dataset. Eight commonly used CNN architectures were trained: EfficientNet-b0, ResNet50, Inception-ResNet-v2, DenseNet121, Inception-v3, VGG19, Xception, and ConvNeXt-Base. The Transformer-based architectures evaluated were ViT-B16, ViT-B32, DeiT-B16, and Swin Base. The confusion matrices obtained for these models are shown in Figure 3.

When examining the confusion matrix results presented in Figure 3, it is observed that all evaluated CNN and ViT architectures generally demonstrate strong discrimination capabilities across all lesion classes. However, this success is more pronounced in the NV (class 6) and VASC (class 7) classes compared to other classes.

Compared to other models, ConvNeXt and Swin Base architectures achieved both high classification performance and a more balanced distribution of correct predictions across dominant and minority classes. This indicates that these modern architectures are more resilient to class imbalance and exhibit a higher generalization capacity in feature extraction.

The performance results of pre-trained CNN and Transformer models are given in Table 3.

When Table 3 is examined, the ConvNeXt-Base model exhibited the highest performance among all CNN models with an accuracy rate of 89.6%. Its macro precision and macro F1-score values were also higher compared to other models. The second model that produced the closest performance metrics to the ConvNeXt-Base model was EfficientNet-b0. The test accuracy achieved by the EfficientNet-b0 model was 88.1%. The model showing the lowest performance among CNN models was VGG19 with 82.5%. The Swin Base Transformer model exhibited the highest performance among ViT-based models with an accuracy rate of 88%. Other performance metrics also achieved the highest values compared to other models. The lowest performance among ViT-based models was achieved by ViT B-16 with a test accuracy of 81%. As a result of these performance analyses, ConvNeXt Base (CNN) and Swin Base (ViT) models were found to offer a more suitable feature extraction structure for the HAM10000 dataset and were determined as the two models to be used in the hybrid architecture.

### 3.2. Results of Proposed Model

The 2048-dimensional feature vector, processed through attention layers obtained from the proposed model, was evaluated on different machine learning classifiers. In this stage, DT, LR, NB, SVM, KNN, and NN methods were applied, and accuracy, macro recall, macro precision, and macro F1-score metrics were calculated for each classifier. The results obtained are given in Table 4.

**Figure 4:**
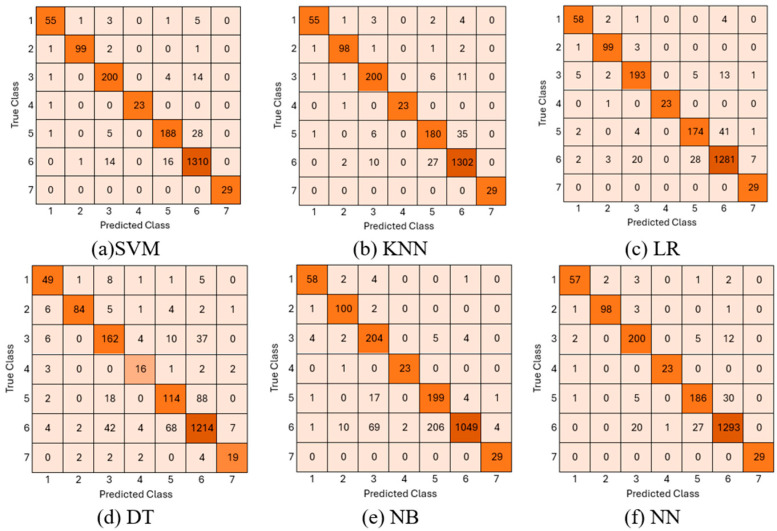
Confusion matrix of the proposed model using ML.

According to the results given in Table 4, the highest success rate was achieved by the SVM classifier with 95.1% accuracy. In addition, the fact that other classifiers generally showed higher performance compared to the basic CNN and ViT architectures reveals the strength of the extracted concatenated features. The confusion matrices of all machine learning methods used are given in Figure 4. In the confusion matrices, the classes are numbered from 1 to 7, and these numbers represent the AKIEC, BCC, BKL, DF, MEL, NV, and VASC classes in the HAM10000 dataset, respectively.

**Figure 5:**
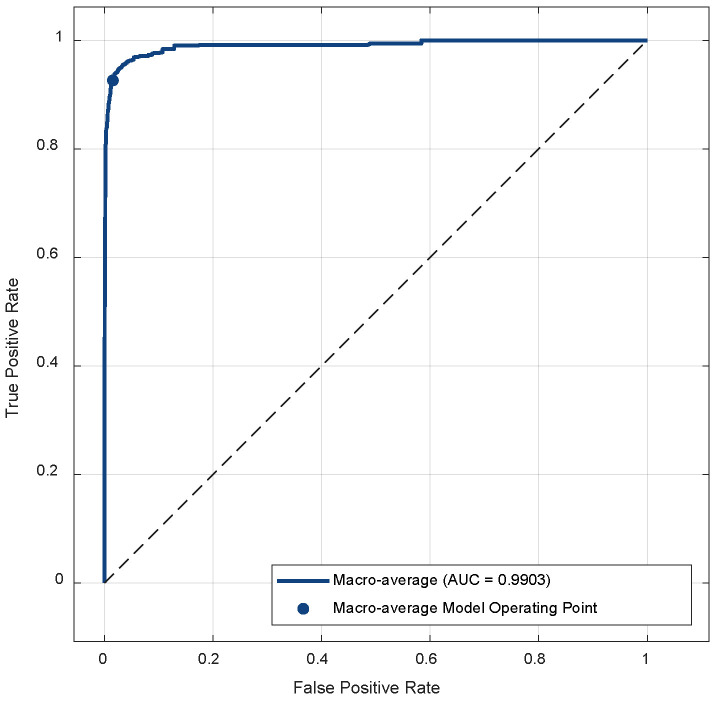
ROC curve of the proposed model.

When Figure 4 is examined, it is seen that, in general, all classifiers exhibit a more balanced performance in both the majority classes and the minority classes thanks to the application of class weights. In particular, the SVM classifier, which has the highest accuracy rate, shows strong discrimination success by correctly predicting 1310 out of 1341 images in the NV class, the most dominant class in the dataset. Performance was also high in the less represented DF and VASC classes. The SVM classifier made only 1 misclassification in the DF class and correctly predicted all samples in the VASC class. The least successful results were obtained in the DT classifier. The ROC curve of the proposed model is presented in Figure 5.

Figure 5 shows the macro-average ROC curve of our proposed model. The curve shown in the graph is close to the upper left corner. This indicates that the model has high classification ability and reliability in all classes. The macro-average AUC value of 0.9903 shows that, despite class imbalance, the model is generally quite successful in distinguishing different class samples.

## 4. Discussion

Skin cancer is one of the most common types of cancer worldwide. The incidence of skin cancer is increasing every day. Melanoma, in particular, has a high mortality rate if not detected in the early stages due to its aggressive course and metastatic potential. Therefore, it has been considered that having systems that can automatically detect skin cancer in the early stages could significantly reduce mortality rates associated with this disease [51]. For this reason, a hybrid model was developed in this study for the early detection of skin cancer types. The literature reports that observer-dependent variability in the evaluation of dermoscopic images can negatively affect diagnostic accuracy. Therefore, the hybrid model developed in our study reduces these limitations caused by the human factor and produces more consistent results. Furthermore, the fact that the obtained performance values are comparable to previous similar studies supports the applicability of the proposed method in terms of clinical decision support systems. The developed model was compared with similar studies in the literature and current pre-trained models. The results show that image-based approaches have a clinically important place in the early diagnosis of skin cancer. A comparative literature review on the subject is presented in Table 5.

Table 5 shows that the proposed model produces more successful results than similar studies in the literature. This indicates that combining the local feature extraction power of CNNs with Vision Transformer and attention mechanisms contributes to obtaining more distinctive representations in multi-class skin cancer classification problems. Furthermore, the imbalance of the dataset was attempted to be corrected using the weight class method. This also contributed to the production of more successful metrics by the proposed model. Although high accuracy values have been reported in some studies with fewer classes, the results obtained on a seven-class and large-scale dataset reveal that the proposed model offers a more suitable and generalizable structure for complex and more-class datasets. The study also has some limitations. One of the biggest limitations is that the dataset used is publicly available. Because the model was not processed on an external dataset obtained from an independent and different source, it poses a limitation in terms of generalizability. In addition, the model needs to be tested with external validation on different devices, different populations, and independent datasets before it goes into clinical application. Another limitation is that the performance in the Melanoma (MEL) class is not perfect, and this carries potential clinical risks. In our study, the number of erroneous predictions obtained for the MEL class is higher than for other classes, and this poses a risk. In a clinical context, it also carries a risk in terms of false-negative melanoma cases, delayed diagnosis, and worsening prognosis. Although class weighting was used in our study, the imbalance of the dataset is another limitation. In future studies, cross-dataset validation on different dermoscopy datasets is planned. In addition, creating a multicenter dataset is among our goals for the research. 

## 5. Conclusions

This study proposes a hybrid model using CNN, Vision Transformer, and Attention mechanisms together for the detection of dermatological images. Experimental results obtained on a seven-class dataset showed that the proposed CNN-ViT-Attention model achieved higher classification accuracy compared to current methods in the literature. The results showed that learning local and global features together improved diagnostic performance, especially in distinguishing complex and multi-class skin lesions. Examination of the performance metrics showed that the proposed model successfully distinguished dermatological images. The proposed model is considered a promising model for clinical decision support systems in the field of dermatology.

## Figures and Tables

**Table 1:** Class-wise distribution of the HAM10000 dataset.

Class	Label	Number of Images
AKIEC	Actinic keratoses	327
BCC	Basal cell carcinoma	514
BKL	Benign keratosis-like	1099
DF	Dermatofibroma	115
MEL	Melanoma	1113
NV	Melanocytic nevi	6705
VASC	Vascular lesions	142

**Table 2:** Implementation details and hyperparameters for fine-tuning.

Parameter	Configuration
Optimization Algorithm	AdamW
Initial Learning Rate	$1\times 10^{-5}$
Learning Rate Scheduler	ReduceLROnPlateau (Factor: 0.1, Patience: 2)
Batch Size	32
Training Epochs	5
Loss Function	Class-Weighted Cross-Entropy
Input Image Size	224 × 224

**Table 3:** Classification performance of pre-trained models.

Model	Accuracy (%)	Macro Recall (%)	Macro Precision (%)	Macro F1 Score (%)
EfficientNet-b0	88.1	79.3	76.8	77.8
Resnet50	86.6	76.5	76.6	75.3
ConvNeXt Base	89.6	79.0	85.6	81.7
Inception_Resnet_v2	87.9	77.5	82.0	78.9
DenseNet 121	88.1	77.4	81.8	79.2
Inception v3	85.9	78.3	73.7	75.2
VGG19	82.5	64.1	67.7	64.5
Xception	88.0	79.7	80.6	79.8
ViT B-16	81.0	60.6	65.6	62.2
ViT B-32	82.0	67.0	69.8	67.9
DeiT B-16	85.8	72.4	74.4	71.1
Swin Base	88.0	76.3	82.1	78.7

**Table 4:** Classification performance of the proposed model using ML.

	Classifiers	Accuracy (%)	Macro Recall (%)	Macro Precision (%)	Macro F1 Score (%)
Proposed Model	DT	82.8	72.1	76.2	74.0
	LR	92.7	91.9	88.7	90.1
	NB	83.0	91.9	81.4	85.2
	SVM	95.1	92.9	95.3	94.0
	KNN	94.2	92.2	94.3	93.2
	NN	94.2	93.0	93.9	93.4

**Table 5:** Literature Review.

Papers	Methods	Number of Classes	Number of Images	Accuracy (%)
Hosny et al. [15]	CNN	3	200	80
Gouda et al. [16]	InceptionV3	2	3533	85.8
Gururaj et al. [17]	DenseNet169	7	10,015	91.2
Kousis et al. [19]	DenseNet169	7	10,015	92.25
Tembhurne et al. [20]	VGG19 + LR	2	3297	93
Wang et al. [21]	VGG	2	25,331	90.67
Balambigai et al. [22]	CNN	7	10,015	77.17
Alwakid et al. [52]	CNN based models	7	10,015	86
Fraiwan and Faouri [53]	CNN architectures	7	10,015	82.9
Alam et al. [54]	S2C-DeLeNet	7	10,015	91.03
Proposed Model	CNN-ViT-Attention	7	10,015	95.1

## Data Availability

The data presented in this study are openly available in https://www.nature.com/articles/sdata2018161 (accessed on 14 October 2025).
